# Prevalence of enteropathogens in children under 15 years of age with special reference to parasites in Kathmandu, Nepal; a cross sectional study

**DOI:** 10.1186/s40064-016-3477-6

**Published:** 2016-10-19

**Authors:** Sachita Dhital, Narayan Dutt Pant, Sanjeev Neupane, Saroj Khatiwada, Bijay Gaire, Jeevan Bahadur Sherchand, Padma Shrestha

**Affiliations:** 1 Department of Microbiology, Kathmandu College of Science and Technology, Kathmandu, Nepal; 2 Department of Microbiology, Grande International Hospital, Dhapasi, Kathmandu, Nepal; 3 Central Department of Microbiology, Tribhuvan University, Kirtipur, Kathmandu, Nepal; 4 Department of Biochemistry, Modern Technical College, Lalitpur, Kathmandu, Nepal; 5 Department of Clinical Microbiology, Institute of Medicine, Tribhuvan University Teaching Hospital, Kathmandu, Nepal

**Keywords:** Enteropathogens, Parasites, Children, Nepal

## Abstract

In developing countries like Nepal, gastrointestinal infections due to various parasites are common causes of morbidity and mortality in children. Present study was carried out from June 2013 to December 2013, among the children (<15 years of age) of Kathmandu Valley. Stool samples were collected from total 600 children (350 from four public schools and slum areas of Kathmandu valley and 250 from pediatric department of Tribhuvan University Teaching Hospital). The main objectives of this study were to investigate the intestinal parasitic infections in children below 15 years of age and their risk factors. However, some bacterial pathogens were also investigated. The overall prevalence of parasitic infections was 29.5 %. The rate of parasitic infections in children from community (39.43 %) was higher than that from hospital (15.6 %; p < 0.05). *Giardia lamblia* was the most common protozoan found to be causing infections in children and among helminths *Ascaris lumbricoides* was the most common worm isolated. Higher rates of parasitic infections were found in children of illiterate parents (38.17 %), children using untreated drinking water (49.77 %) and children having habit of consuming raw vegetables (31.50 %; p < 0.05). The present study indicated that the rate of infections due to enteropathogenic parasites was high among children of Nepal.

## Background

 Diarrheal diseases are the important causes of morbidity and mortality among young children in developing countries; with significant numbers of cases being attributable to parasites (Sherchand et al. 2009). Their prevalence depends upon various socio-economic factors such as hygiene, availability of clean drinking water, poverty and education (Tandukar et al. 2013). Diarrheal diseases mainly affect young children, diminishing cognitive function (Niehaus et al. 2002) and are the second major leading causes of childhood mortality (World Health Organization 2013). Diarrheal diseases account for large number of child deaths in Asia, Latin America and Africa (Uga et al. 2004).

Diarrheal diseases are the major causes of deaths in developing countries (Bansal et al. 2004). Nepal is a least developed country with various parasitic infections, being important causes of morbidity and mortality (Uga et al. 2004). So, the proper identification of etiological agent of the disease is extremely necessary to provide an effective treatment.

In this study, we investigated the involvement of parasites in causing diarrheal diseases in children below 15 years of age. We also investigated some bacterial pathogens. Further, we studied the correlation between rate of gastrointestinal parasitic infections with many factors like food habit, source of water used and education of the parents. This study will help to explore actual current status of diarrheal diseases caused by parasites in children of Nepal. The study will be valuable for policy makers to make policies regarding case finding, diagnosis, management, prevention and control of intestinal parasitic infections in children.

## Methods

A cross section study was carried among children (below 15 years of age), from June 2013 to December 2013. Total of 600 stool samples (250 samples from children visiting pediatric department of Tribhuvan University Teaching Hospital and 350 samples from children from four public schools and slum areas of Kathmandu valley) were collected. Children with gastrointestinal discomfort were only enrolled in the study and those who already had got antimicrobials were excluded from the study. Patient’s consent form was used to obtain written informed consent from all the patient’s guardians. Before commencing of the study, the research protocol was approved by Tribhuvan University Teaching Hospital and Kathmandu College of Science and Technology.

### Sample collection

Each subject was provided with a clean, dry, disinfectant free, wide mouthed container and asked to collect about 20 g of stool specimen into the container. They were cautioned not to contaminate the stool with water and urine. The collected stool samples were immediately brought to the Public Health Research Laboratory, Institute of Medicine, Kathmandu, Nepal and processed as follows:

### Macroscopic examination

The direct visual examination of each sample was done for the color, consistency, presence of blood, presence of mucus and presence of adult worms or worm segments.

### Microscopic examination

All the samples were subjected to concentration (by formal-ether sedimentation method and Sheather’s sucrose floatation technique). Microscopic examination was done by saline wet mount, iodine wet mount and modified acid-fast staining technique. The mounted slides were examined under low power (10X) followed by high power (40X) and acid-fast stained smears were examined using oil immersion (100X). Parasites were identified with the help of their morphology, motility and staining reaction.

### Sporulation of *Cyclospora cayetanensis* oocysts

*Cyclospora cayetanensis* oocysts are excreted unsporulated in the feces. Specific identification of this coccidian parasite can be established by stimulating its sporulation and subsequent finding of two sporocysts within each oocyst of the parasite. For the enhancement of sporulation, about 2 g of stool sample was mixed with about 5 ml of 2.5 % potassium dichromate solution and incubated at room temperature for 15 days. Sporulation was confirmed by light microscopy by observing two sporocysts in each oocyst.

### Culturing of stool samples on differential, selective and enrichment medium for isolation of bacterial pathogens

The stool samples were cultured on Mac-Conkey agar for differentiation of gram negative organisms. For the enrichment of *Vibrio cholerae* alkaline peptone water was used and selenite F broth was used for enrichment of *Salmonella* and *Shigella*. Thiosulfate citrate bile salts sucrose (TCBS) agar was used for isolation of *V. cholerae* and Salmonella–Shigella (SS) agar was used for the isolation of *Salmonella* and *Shigella*. The stool samples were directly inoculated in Mac-Conkey agar. But before inoculating into TCBS the stool samples were inoculated into alkaline peptone water and incubated for 24 h at 37 °C. Similarly, before inoculating into SS agar the stool samples were inoculated into selenite F broth and incubated for 24 h at 37 °C.

### Observations of culture plates

Each plate was observed after 48 h of aerobic incubation at 37 °C for the growth of characteristic bacteria. Mac-Conkey agar was observed for the growth of gram negative organisms. Salmonella–Shigella agar was observed for typical colonies of *Salmonella* and *Shigella*. Similarly, in TCBS agar colonies typical of *V. cholerae* were sought.

### Identification of the bacterial isolates

The bacterial isolates were identified with the help of colony morphology, conventional biochemical testing and serotyping. For serotyping of *Salmonella*, specific antisera (Denka Seiken Co. Ltd, Tokyo, Japan) was used.

### Antibiotic susceptibility testing

The bacterial isolates were subjected to antibiotic susceptibility testing by Kirby-Bauer disc diffusion technique according to the Clinical and Laboratory Standards institute (CLSI) guidelines (Clinical and Laboratory Standards Institute 2012).

### Quality control

For quality control of biochemical testing, purity plate (the bacterial media plate which is divided into two halves and the bacterial inoculums before and after performing biochemical tests are inoculated into two different halves, so as to see if there was contamination during processing) was used and for standardization of the antimicrobial susceptibility testing, control strain *Escherichia coli* (ATCC 25922) was used.

### Data processing and analysis

The data obtained were entered into MS excel and analyzed using SPSS version 11.0. Chi square test was applied and *p* value <0.05 was taken as significant.

## Results

### Distribution of parasites in samples collected from community and hospital

Out of total 600 samples, 177 (29.5 %) had parasites. Among total 350 stool samples collected from community, the parasites were detected in 138 (39.43 %) samples. Similarly, among 250 samples collected from hospital, 39 (15.6 %) samples were found to contain parasites and the difference was statistically significant (p < 0.05).

### Age wise distribution of parasitic infections

38.85 % of the patients in the age group 3–6 were found to be infected with parasites followed by patients in the age group 9–12 (32.19 %; Table 1).

**Table 1:** Age wise distribution of parasitic infections

Age (years)	Male	Female	Total	Total positive	Percentage (%)
<3	44	37	81	17	20.99
3–6	97	60	157	61	38.85
6–9	54	32	86	21	24.42
9–12	78	68	146	47	32.19
12–15	73	57	130	31	23.85
Total	346	254	600	177	

### Site wise distribution of parasitic infections

In hospital, rates of monoparasitosis and multiparasitosis were 12.4  and 3.2 % respectively. In community, 33.14 % had monoparasitosis and 6.29 % had multiparasitosis. In community, protozoa were isolated from 36.57 % of the patients, helminths were isolated from 2.29 % of the patients and both protozoa and helminths were isolated from 0.57 % of the patients. Similarly, in the hospital, the protozoa were isolated from 14 % of the patients, helminths were isolated from 1.2 % of the patients and both protozoa and helminths were isolated from 0.4 % of the patients. Out of 177 parasite positive samples, protozoa were isolated from 92.09 % of the samples, helminths were isolated from 6.21 % of the samples and both helminths and protozoa were isolated from 1.69 % of the samples. Among protozoa, *Giardia lamblia* was most common followed by *Entamoeba histolytica*. Among helminths, *Ascaris lumbricoides* was most common followed by *Hymenolepis nana* (Table 2).

**Table 2:** Site wise distribution of parasitic infections

Parasites	Sites		Total
	Community	Hospital	
*Entamoeba histolytica* (EH)	42	14	56
*Giardia lamblia* (GL)	51	11	62
*Blastocystis hominis* (BH)	3	1	4
*Cyclospora* spp.	5	1	6
*Cryptosporidium* spp.	5	0	5
*Entamoeba coli*	2	1	3
Total single protozoa	108	28	136
EH + GL	5	2	7
EH + *Cyclospora* spp.	2	1	3
GL + *Cyclospora* spp.	3	2	5
EH + BH	2	0	2
EH + *Cryptosporidium* spp.	2	1	3
GL + *Cryptosporidium* spp.	3	0	3
EH + *Entamoeba coli*	2	1	3
*Entamoeba coli* +BH + *Cryptosporidium* spp.	1	0	1
Total multiple protozoa	20	7	27
*Ascaris lumbricoides*	4	2	6
*Hymenolepis nana*	2	1	3
*Trichuris trichiura*	1	0	1
*Schistosoma* spp.	1	0	1
Total single helminths	8	3	11
*GL* + *Hymenolepis nana*	1	1	2
*EH* + *Trichuris trichiura*	1	0	1
Total helminths + protozoa	2	1	3
Total parasite positive cases	138	39	177

### Month wise distribution of parasites

The rate of detection of parasites in month of August was (53/127) 41.73 % followed by that in July (51/152) 33.55 % (Table 3).

**Table 3:** Month wise distribution of parasites

Month	Total processed sample	Total positive cases	Positive (%)
July	152	51	33.55
August	127	53	41.73
September	114	30	26.32
October	116	29	25
November	44	5	11.36
December	47	9	19.15
Total	600	177	

### Distribution of parasite positive cases according to parent’s education

Among 351 children having illiterate parents, 134 (38.18 %) had parasitic infections and among 249 children having literate parents, 43 (17.27 %) had parasitic infections. Statistically, there was significant association between parent’s education and parasitic infections in children (p < 0.05).

### Parasitic infections in relation to food habit

Out of 600 children, 529 were non vegetarian and 71 were vegetarian. Among vegetarian, 21 were found to be infected with parasites and among non vegetarian, 156 were infected. Statistically, there was no significant association between food habit and parasitic infections in children.

### Distribution of parasite positive cases on the basis of sources of drinking water used by the patients

29.86 % (126/422) of the patients using tap water as drinking water, 30.77 % (40/130) of the patients using well water as drinking water and 22.92 % (11/48) of the patients using jar water (drinking water sterilized and sealed in jar) as drinking water were found to be infected with parasites. Statistically, there was no association between parasitic infections and sources of drinking water used.

### Distribution of parasite positive cases on the basis of types of drinking water used by the patients

49.77 % of the children, who drink untreated water and 18.58 % of the children drinking filtered water had parasitic infections. Statistically, there was significant association between types of drinking water and parasitic infections in children (p < 0.05; Table 4).

**Table 4:** Distribution of parasite positive cases on the basis of types of drinking water used by the patients

Water type	Total	Positive (%)	p value
Filtered	323	60 (18.58)	<0.05
Chlorinated	38	7 (18.42)	<0.05
Boiled	20	1 (5)	<0.05
Untreated	219	109 (49.77)	<0.05
Total	600	177	

### Distribution of parasite positive cases on the basis of raw vegetable consumption

31.51 % (167/530) of the raw vegetable consumers and 14.29 % (10/70) of raw vegetable non consumers had parasitic infections. There was statistical association between parasitic infections and raw vegetable consuming habit in children (p < 0.05).

### Distribution of bacterial enteropathogens

Out of total 600 stool samples collected, pathogenic bacteria were isolated from 51 samples. Of which, 29 samples contained *Shigella* spp. and 22 samples contained *Salmonella* spp.

### Distribution of different species of *Shigella* and different serotypes of *Salmonella* spp.

Among 29 *Shigella* spp. isolates, *Shigella flexneri* was the most common species and among 22 *Salmonella* spp. isolates, *Salmonella* Paratyphi B was most common (Table 5).

**Table 5:** Frequency distribution of species of *Shigella* spp. and serotypes of *Salmonella* spp.

Bacterial enteropathogens	Total
*Shigella dysenteriae*	4
*Shigella flexneri*	12
*Shigella boydii*	8
*Shigella sonnei*	5
Total *Shigella* spp.	29
*Salmonella Typhi*	6
*Salmonella Paratyphi A*	4
*Salmonella Paratyphi B*	12
Total *Salmonella* spp.	22
Total	51

### Antibiotic susceptibility patterns of *Shigella* spp. and *Salmonella* spp.

The *Shigella* spp. were found to be most susceptible to gentamicin (86.21 %) followed by ciprofloxacin (82.76 %). Similarly, *Salmonella* spp. were found to be most susceptible to tetracycline (90.91 %) followed by chloramphenicol (86.36 %; Table 6).

**Table 6:** Antibiotic susceptibility patterns of *Shigella* spp. and *Salmonella* spp.

Antibiotics	*Shigella* spp. (n = 29)		*Salmonella* spp. (n = 22)	
	Sensitive (%)	Resistant (%)	Sensitive (%)	Resistant (%)
Ciprofloxacin	24 (82.76)	5 (17.24)	16 (72.73)	6 (27.27)
Gentamicin	25 (86.21)	4 (13.79)	–	–
Cotrimoxazole	16 (55.17)	13 (44.83)	17 (77.27)	5 (22.73)
Ampicillin	13 (44.83)	16 (55.17)	10 (45.45)	12 (54.55)
Ofloxacin	–	–	16 (72.73)	6 (27.27)
Nalidixic acid	14 (48.28)	15 (51.72)	–	–
Tetracycline	17 (58.62)	12 (41.38)	20 (90.91)	2 (9.09)
Cefotaxime	17 (58.62)	12 (41.38)	15 (68.18)	7 (31.82)
Chloramphenicol	–	–	19 (86.36)	3 (13.64)

## Discussion

The result of our study was similar with finding of the study done in similar setting in Kathmandu, Nepal by Lama and Sherchan, who reported the rate of intestinal parasitic infection to be 104/285 (36.5 %; Lama and Sherchan 2008). Conditions most frequently associated with high prevalence of intestinal parasitic infections include personal hygiene and microbiological quality of drinking water.

Akinbo et al. (2011) reported high prevalence of parasitic infections during rainy season. Due to the practice of open defecation near water sources, in rainy season the feces may be washed away into the source of drinking water (Mbae et al. 2013). Further the sewage water and over flooded water may contaminate the drinking water supply and if such water is used without pretreatment, the chances of infection will be high. Protozoa dominating the parasitic helminths in our study was in agreement with the previous finding by Thapa Magar et al. (2011). Similar to our finding, high prevalence of *G. lamblia* followed by *E. histolytica* was also reported by other researchers in Nepal (Chandrashekhar et al. 2005; Gyawali et al. 2009; Khadka et al. 2013). Among all intestinal protozoan parasites, *G. lamblia* is the most predominant parasite among school-age children and the children of this age group carry higher parasitic burden than adults (Cook et al. 2009).

In our study helminth infections were less prevalent as compared to the protozoal infections, but Chandrashekhar et al. (2005), reported a higher prevalence of soil transmitted helminths in Nepal. Helminth infections are particularly associated with iron and vitamin-A deficiencies and after anti-helminthic drug administration, improvement in iron status and vitamin-A absorption is seen (Shrestha and Sharma 2012). The lower prevalence of helminth infections in children seen in present study could possibly be explained by periodic campaign of deworming conducted by ministry of health (Tandukar et al. 2013).

Statistically, significant difference was found between prevalence of intestinal parasites in the children of literate parents and that in the children of illiterate parents. This may be due to lack of knowledge about the parasites, their way of transmission and preventive measures among illiterate parents. Literate parents are more likely to give correct care to their children when they have diarrhea and also more likely to seek medical care for a child with diarrhea. So, to minimize rate of infection, it is suggested to increase the numbers of awareness programs regarding the mode of transmission of intestinal parasites and their preventive measures.

Present study showed that the boiled water was more appropriate for drinking purpose than raw, filtered and chlorinated water. The main reason of this is, boiling of water kills the microorganisms and prevents transmission of infection. Due to heavily contaminated drinking water sources of Kathmandu valley, methods other than boiling used for disinfection of drinking water may not have been as effective as boiling (Prasai et al. 2007).

There was statistical association between parasitic infections and raw vegetable consuming habit. High rate of parasitic infections in raw vegetable consumers may be due to use of fecal contaminated water for irrigation of vegetables or contaminated hands of food handlers. Moreover, in Nepal vegetables available in the markets are rinsed into highly contaminated water of ponds or rivers in order to wash and clean the soil.

Globally, *Salmonella* spp. and *Shigella* spp. remain as major causes of acute enteric infections (Abu Elamreen et al. 2008). Shigellosis is one of the most common public health problems in developing countries and about 80 % of infections due to *Shigella* spp. occur in children under 10 years of age (Shah et al. 2012). Shigellosis is extremely contagious disease, prevalent among crowded and poor population (Nicolas et al. 2007).

Similar to our study, *Shigella flexneri* was the most common species of *Shigella* isolated in Nepal by Khan et al. (2013) and Kanskar et al. (2007). Higher resistance of *Shigella* spp. than in our study was observed for trimethoprim-sulphamethoxazole, ampicillin, nalidixic acid, and ciprofloxacin in study done by Khan et al. (2013). But rate of resistance to gentamicin was similar to our study (Khan et al. 2013).

As in our study Pokharel et al. (2009) reported, all serotypes of *Salmonella* isolated from children from Kathmandu, Nepal to be Typhi or Paratyphi. This may be due to the endemicity of these serotypes in Kathmandu. Similar rate of susceptibility of *Salmonella* spp. (isolated from stool) to chloramphenicol and tetracycline, as in our study was also found by Ansari et al. (2012). But the antimicrobial treatment of diarrhea is only recommended in selected cases as those caused by *Shigella* spp. infections or invasive serotypes of *Salmonella* spp.

### Limitations of the study

Since this study was conducted in low income country, due to lack of fund we could not use molecular methods to confirm our results. Further, due to availability of the limited resources we could not include a wide range of pathogens like diarrheagenic *Escherichia coli*, *Campylobacter* spp.*, Yersinia enterocolitica*, *Clostridium difficile* and viruses in our study.

## Conclusions

The present study indicated that the prevalence of infections due to enteropathogenic parasites was high among children of Nepal. Further, there was association between rate of infection by intestinal parasites among children and parent’s education, types of drinking water (filtered, chlorinated, boiled, untreated) used and habit of raw vegetable consumption.

## References

[CR1] Abu Elamreen FH, Sharif FA, Deeb JE (2008). Isolation and antibiotic susceptibility of Salmonella and Shigella strains isolated from children in Gaza, Palestine from 1999 to 2006. J Gastroenterol Hepatol.

[CR2] Akinbo FO, Okaka CE, Omoregie R (2011). Seasonal variation of intestinal parasitic infection among HIV-positive patients in Benin City, Nigeria. Ethiop J Health Sci.

[CR3] Ansari S, Sherchand JB, Parajuli K, Mishra SK, Dahal RK, Shrestha S (2012). Bacterial etiology of acute diarrhea in children under five years of age. J Nepal Health Res Counc.

[CR4] Bansal D, Sehgal R, Bhatti HPL, Shrivastav SK, Khurana S, Mahajan RC (2004). Intestinal parasites and intrafamilial incidence in a low socioeconomic area of Chandigarh (North India). Nepal Med Coll J.

[CR5] Chandrashekhar TS, Joshi HS, Gurung M, Subba SH, Rana MS, Shivananda PG (2005). Prevalence and distribution of intestinal parasitic infestations among school children in Kaski District, Western Nepal. J Biomed Sci.

[CR6] Clinical and Laboratory Standards Institute (2012) CLSI Document M100-S22. Performance standards for antimicrobial susceptibility testing: twenty second informational, Supplement ed. CLSI, Wayne

[CR7] Cook DM, Swanson RC, Eggett DL, Booth GM (2009). A retrospective analysis of prevalence of gastrointestinal parasites among school children in the Palajunoj Valley of Guatemala. J Health Popul Nutr.

[CR8] Gyawali N, Amatya R, Nepal HP (2009). Intestinal parasitosis in school going children of Dharan Municipality, Nepal. Trop Gastroenterol.

[CR9] Kansakar P, Malla S, Ghimire GR (2007). Shigella isolates of Nepal: changes in the incidence of shigella subgroups and trends of antimicrobial susceptibility pattern. Kathmandu Univ Med J.

[CR10] Khadka KS, Kaphle HP, Gurung K, Manoj Sigdel M (2013). Study of intestinal parasitosis among school going children in Pokhara, Nepal. J Health Allied Sci.

[CR11] Khan S, Singh P, Asthana A, Ansari M (2013). Magnitude of drug resistant shigellosis in Nepalese patients. Iran J Microbiol.

[CR12] Lama C, Sherchan JB (2008). Enteropathogens associated diarrhea in hospitalized patients of children’s hospital, Kathmandu. J Nepal Health Res Council.

[CR13] Mbae CK, Nokes DJ, Mulinge E, Nyambura J, Waruru A, Kariuki S (2013). Intestinal parasitic infections in children presenting with diarrhoea in outpatient and inpatient settings in an informal settlement of Nairobi, Kenya. BMC Infect Dis.

[CR14] Nicolas X, Granier H, Le Guen P (2007). Shigellosis or bacillary dysentery. Presse Med.

[CR15] Niehaus MD, Moore SR, Patrick PD, Derr LL, Lorntz B, Lima AA (2002). Early childhood diarrhea is associated with diminished cognitive function 4 to 7 years later in children in a northeast Brazilian Shantytown. Am J Trop Med Hyg.

[CR16] Pokharel M, Sherchand JB, Upreti HC, Katuwal A, Gauchan P (2009). A perspective study on the etiology of Diarrhea in children less than 12 years of age attending Kanti Children’s Hospital. J Nepal Paediatr Soc.

[CR17] Prasai T, Lekhak B, Joshi DR, Baral MP (2007). Microbiological analysis of drinking water of Kathmandu valley. Sci World.

[CR18] Shah BK, Sharma S, Shakya S, Upadhyay BP (2012). Cholera, Shigellosis and Salmonellosis incidence among the people of some Districts of Nepal. Nepal J Sci Technol.

[CR19] Sherchand JB, Yokoo M, Sherchand O, Pant AR, Nakagomi O (2009). Burden of enteropathogens associated diarrheal diseases in children hospital, Nepal. Sci World.

[CR20] Shrestha AKCN, Sharma R (2012). Prevalence of intestinal parasitosis among school children in Baglung District of Western Nepal. Kathmandu Univ Med J.

[CR21] Tandukar S, Ansari S, Adhikari N, Shrestha A, Gautam J, Sharma B (2013). Intestinal parasitosis in school children of Lalitpur district of Nepal. BMC Res Notes.

[CR22] Thapa Magar D, Rai SK, Lekhak B, Rai KR (2011). Study of parasitic infection among children of Sukumbasi Basti in Kathmandu valley. Nepal Med Coll J.

[CR23] Uga S, Rai SK, Kimura K, Ganesh R, Kimura D, Wakasugi M (2004). Parasites detected from diarrheal stool samples collected in Nepal. Southeast Asian J Trop Med Public Health.

[CR24] World Health Organization (2013) Diarrhoeal disease Fact sheet

